# Experimental infection of tigerfish (*Hydrocynus vittatus*) and African sharp tooth catfish (*Clarias gariepinus*) with *Trichinella zimbabwensis*

**DOI:** 10.4102/ojvr.v87i1.1876

**Published:** 2020-11-05

**Authors:** Louis J. la Grange, Samson Mukaratirwa

**Affiliations:** 1 Department of Agriculture, Rural Development, Land and Environmental Affairs, Chief Directorate Veterinary Services, Veterinary Public Health, Mbombela, South Africa; 2 School of Life Sciences, College of Agriculture, Science and Engineering, University of KwaZulu-Natal, Durban, South Africa; 3 One Health Center for Zoonoses and Tropical Veterinary Medicine, School of Veterinary Medicine, Ross University, Basseterre, Saint Kitts and Nevis

**Keywords:** *Clarias gariepinus*, *Hydrocynus vittatus*, tiger fish, infectivity, *Trichinella zimbabwensis*

## Abstract

*Trichinella zimbabwensis* naturally infects a variety of reptilian and wild mammalian hosts in South Africa. Attempts have been made to experimentally infect piranha fish with *T. zimbabwensis* and *T. papuae* without success. Tigerfish (*Hydrocynus vittatus*) and African sharp tooth catfish (*Clarias gariepinus*) are accomplished predators cohabiting with Nile crocodiles (*Crocodylus niloticus*) and Nile monitor lizards (*Varanus niloticus*) in southern Africa and are natural hosts of *T. zimbabwensis*. To assess the infectivity of *T. zimbabwensis* to these two hosts, 24 African sharp tooth catfish (mean live weight 581.75 ± 249.71 g) randomly divided into 5 groups were experimentally infected with 1.0 ± 0.34 *T. zimbabwensis* larvae per gram (lpg) of fish. Forty-one tigerfish (mean live weight 298.6 ± 99.3 g) were randomly divided for three separate trials. An additional 7 tigerfish were assessed for the presence of natural infection as controls. Results showed no adult worms or larvae of *T. zimbabwensis* in the gastrointestinal tract and body cavities of catfish sacrificed at day 1, 2 and 7 post-infection (p.i.). Two tigerfish from one experimental group yielded 0.1 lpg and 0.02 lpg of muscle tissue at day 26 p.i. and 28 p.i., respectively. No adult worms or larvae were detected in the fish from the remaining groups sacrificed at day 7, 21, 28, 33 and 35 p.i. and from the control group. Results from this study suggest that tigerfish could sustain *T. zimbabwensis* under specific yet unknown circumstances.

## Introduction

The genus *Trichinella* belongs to the family Trichinellidae and the Order Trichuridae, class Nematode (Pozio et al. 2009). *Trichinella* establishes in the host when larvae in raw or undercooked meat are consumed (Dupoy-Camet 2000). Parasites in the genus complete both intermediate and definitive life cycle stages in a single host. Larvae are intracellular parasites of striated muscle tissue and adult nematodes parasitise the intestinal epithelium (Gottstein, Pozio & Nöckler 2009).

*Trichinella zimbabwensis* has the widest host range and is the most prevalent of the three known *Trichinella* spp. (i.e. *T. nelsoni, T. zimbabwensis* and *Trichinella* T8) in the Greater Kruger National Park (GKNP). It naturally infects various reptilian and wild mammalian hosts in South Africa (La Grange & Mukaratirwa 2020; Mukaratirwa et al. 2019). Amongst the confirmed natural hosts, the highest prevalence has been recorded in Nile crocodile (*Crocodylus niloticus*) (La Grange, Govender & Mukaratirwa 2013).

In the last two decades, significant strides were made in identifying natural hosts of *Trichinella* spp. in the GKNP (Mukaratirwa et al. 2019) and continued surveillance to identify additional hosts for *T. zimbabwensis* is still required (La Grange & Mukaratirwa 2020; La Grange et al. 2013). Previous experimental studies involving fish elsewhere showed some *Trichinella* species are able to survive for short periods in the gut (Moretti et al. 1997; Tomašovičová 1981), suggesting some tropical fish to act as definitive or paratenic hosts for the parasite.

Pienaar (1968) reported the presence of 46 fish species, including predatory species such as African sharp tooth catfish (*Clarias gariepinus*), tigerfish (*Hydrocynus vittatus*), Madagascar mottled eel (*Anguilla marmorata*) and common long fin eel (*A. mossambicus*) in the KNP.

Catfish are accomplished scavengers and predators, and consume a variety of food ranging from plankton to small mammals (Bruton 1979; Skelton 2001). Tigerfish are apex predators that occupy the same trophic level as Nile crocodiles where they cohabit (Roux et al. 2018). The feeding behaviour of these fish makes them potential hosts of *T. zimbabwensis*.

This study aimed at assessing the infectivity of *T. zimbabwensis* to catfish and tigerfish which cohabit natural environments with Nile crocodile, a known natural host of the parasite.

## Materials and methods

Twenty-four African sharp tooth catfish from a research breeding and holding facility of the University of Limpopo, South Africa were housed outdoors in moveable ponds at a research facility at Tipperary farm, Nelspruit, South Africa. They were fed commercial koi pellets daily (Aqua Plus, Midfeeds, South Africa).

Tigerfish were collected from two sites along the Komati river (31˚55’07.1’’E, 25˚29’41.4’’S and 31˚58’02.3’’E, 25˚29’12.8’’S) and housed indoors on a private farm near one of the collection sites. Ponds were equipped with an inline filtration system and submersible pump (6500 L/h). A water heater (UltraZap, 3 kw) was additionally installed during two of the trials to maintain average water temperature within the fish’s natural range.

Water temperature was recorded for 4 h intervals in the ponds and Komati River using temperature loggers (Hobo Water temp pro V2, Onsetcomp).

*Trichinella zimbabwensis* larvae, previously derived from a Nile crocodile, were maintained in a colony of rats housed at the Biological Resource Unit (BRU) at the Westville Campus of the University of KwaZulu-Natal. Infected rats were euthanised and larvae isolated using artificial muscle digestion (Nöckler & Kapel 2007).

Additional *Trichinella*-infected muscle tissue was collected and mixed into a homogenous, pooled sample containing 50 larvae per gram (lpg) to infect catfish via natural feeding.

Ten catfish were infected using an orogastric tube (Table 1). Fish in Group 1 (*n* = 5) were sedated with 0.3 mL/l of 2-phenoxyethanol in water. Once stage 2 anaesthesia was reached (Gardner 2017), the fish were individually infected with approximately 500 larvae administered via a canine urinary catheter (Sovereign^®^, French Gauge 8, Sherwood Medical, St. Louis, United States of America). The procedure was repeated for Group 2 (*n* = 5) but without anaesthetic, to rule out possible negative effects of anaesthetic on the establishment and/or development of *T. zimbabwbensis* larvae.

**TABLE 1:** Results from experimental infection of African sharp tooth catfish (*Clarias gariepinus*) with *Trichinella zimbabwensis.*

Group	N	M/F	Weight (g) $\bar{\text{x}}$ ± SD	Infection method	Lpg of fish $\bar{\text{x}}$ ± SD	T °C $\bar{\text{x}}$ ± SD	Day (p.i.)	Infection results		
								Body cavity	Intestines	Muscle
Group 1	5	3/0	354.9 ± 75.46	Orogastric (anaesthesia)	1.26 ± 0.58	22.47 ± 1.28	7	Neg	Neg	Neg
Group 2	5	5/0	531.0 ± 160.3	Orogastric (no anaesthesia)	1.02 ± 0.29	15.59 ± 0.50	7	Neg	Neg	Neg
Group 3	5	5/0	568.0 ± 109.7	Gelatin capsules (anaesthesia)	0.92 ± 0.20	15.49 ± 0.36	2	Neg	Neg	Neg
Group 4	5	3/2	499.6 ± 74.1	Gelatin capsules (no anaesthesia)	1.02 ± 0.14	15.55 ± 0.41	1	Neg	Neg	Neg
Group 5	4	4/0	1048.0 ± 137.8	Natural Feeding	1.00 ± 0.11	21.58 ± 1.36	2	Neg	Neg	Neg

M/F, male/female; SD, standard deviation; Neg, negative for infection; p.i., post-infection; lpg, larvae per gram.

An additional 10 fish were infected using blank gelatin capsules (size 0, Medisurge, South Africa). Capsules containing approximately 500 larvae were administered orally. Group 3 (*n* = 5) was infected with, and Group 4 (*n* = 5), without anaesthesia (Table 1).

Four fish (Group 5) were infected through natural feeding with approximately 1000 larvae/fish (Table 1). Ten grams of infective tissue (50 lpg) was used to prepare infective boluses (approximately 500 larvae). Two boluses were offered to each fish on the day of infection and were readily consumed by the fish.

Fresh chicken hearts (Mikon Farming abattoir, South Africa) were ‘loaded’ with a 2 mL solution of infective material (approximately 300 larvae/mL) using a 3 mL syringe and 25 gauge needle. Each fish received a single infected chicken heart. Three separate trials were conducted (Table 2).

**TABLE 2:** Results from experimental infection of tigerfish (*Hydrocynus vittatus*) with *Trichinella zimbabwensis.*

Trial (T)/Group (G)	N	M/F	Weight(g) $\bar{\text{x}}$ ± SD	Lpg of fish $\bar{\text{x}}$ ± SD	T (˚C) $\bar{\text{x}}$ ± SD	Day (p.i.)	No tested	Infection results		
								Body cavity	Intestines	Muscle
T_1_G_1_	8	5/3	301.3 ± 95.7	2.2 ± 0.9	25.6 ± 1.6	28	4	Neg	Neg	0.02†; 0.1††lpg
T_2_G_1_	5	4/1	265.4 ± 103.5	2.7 ± 1.1	25.1 ± 1.3	28	5	Neg	Neg	Neg
T_2_G_2_	6	4/1	242.0 ± 105.4	2.9 ± 1.2	24.9 ± 1.2	35	6	Neg	Neg	Neg
T_3_G_1_	5	4/1	356.3 ± 71.9	0.6 ± 0.1	26.9 ± 0.4	7	5	Neg	Neg	Neg
T_3_G_2_	5	1/4	305.7 ± 103.3	2.2 ± 0.9	26.4 ± 0.9	21	5	Neg	Neg	Neg
T_3_G_3_	5	3/2	330.5 ± 56.7	1.9 ± 0.3	26.4 ± 1.6	33	5	Neg	Neg	Neg

Results for control fish (*n* = 7) not included in the above results. Controls were negative.

T_1_G_1_, Trial 1 Group 1; T_1_G_2_, Trial Group 2; T_3_G_1_, Trial 3 Group 1; T_3_G_2_, Trial 3 Group 2; T_3_G_3_, Trial 3 Group 3.

M/F, male/female; SD, standard deviation; Neg, negative for infection; p.i., post-infection; lpg, larvae per gram.

† , Muscle larvae observed on day 28 p.i.

†† , Muscle larvae observed on day 26 *p*.i.

Tigerfish intended for trial 1 (T_1_) (*n* =1), trial 2 (T_2_) (*n* = 3) and trial 3 (T_3_) (*n* = 3) died during the respective first weeks of acclimation because of stress, preserved (frozen) and retained as a control group.

Fish were euthanised on day 1, 2 and 7 p.i. (catfish) and 7, 21, 28, 33 and 35 p.i. (tigerfish) using 0.6 mL/l of 2-phenoxyethanol. Fish were allowed to reach stage 4 anaesthesia (Gardner 2017) before being decapitated.

Each fish was dissected ventrally and opened lengthwise from the cloaca to the head. The body cavity was rinsed with 0.9% saline solution and the wash collected in a glass Petri dish. The small intestines and stomach were separated into specimen dishes containing 0.9% saline solution (Justine, Briand & Bray 2012), cut open and the mucosa carefully scraped. The contents and the wash liquid were examined for the presence of *T. zimbabwensis* larvae and/or adults. Additionally, a representative sample of the whole body comprising 100 g of muscle tissue was collected, artificially digested (Nöckler & Kapel 2007) and examined for the presence of *T. zimbabwensis* larvae.

The average weight ± standard deviation (SD) was expressed per group. Water temperature was expressed as average daily water temperature ± SD for each group. Mean temperature of the ponds during each trial was compared with the mean temperature of the river during the corresponding time frame using student T-tests.

### Ethical consideration

The study protocol was approved by the Animal Research Ethics Committee of the University of KwaZulu-Natal (Ref: AREC/029/016M and 030/019D).

## Results and discussion

Results from the experimental trials are shown in Tables 1 and 2. No larvae or adults of *T. zimbabwensis* were observed in the gastrointestinal tract, body cavities and muscle tissue of catfish infected using different modes at day 1, 2 or 7 p.i. (Table 1). Because of prolonged national power failures during the last 2 weeks of T_1_G_1_, four tigerfish died at day 23 p.i and two at day 26 p.i. from oxygen depletion of the water. The two surviving fish were euthanised at day 28 p.i.

Analysis of the two tigerfish that died on day 26 p.i. yielded a total of 10 dead larvae (1 lpg of muscle) from one fish. Of the two that were euthanised one fish yielded a live larva (0.02 lpg) in muscle tissue. The low number of larvae recovered is in agreement with findings from studies involving *T. britovi* (Moretti et al. 1997) and *T. spiralis* and *T. pseudospiralis* (Tomašovičová 1981). No larvae or adult parasites were observed in the body cavity, stomach or gastrointestinal tract of fish euthanised at day 7, 28, 33 or 35 p.i.

The minimum infective larval dose required in a specific host is dependant on both parasite- and host characteristics (Gottstein et al. 2009). Previous studies involving fish experimentally infected with *T. pseudospiralis* and *T. spiralis* (Tomašovičová 1981), *T. britovi* (Moretti et al. 1997) and *T. zimbabwensis* and *T. papuae* (Pozio & La Rosa 2005) were reported on varying dosages of *Trichinella* larvae administered to fish and no mention was made of the live weight of fish used. The lack of data in respect of the weight of fish and larvae per gram of fish administered in previous studies precludes a comparison to assess the minimum infective dosage needed for *T. zimbabwensis* to establish in fish.

In an experimental study involving *T. zimbabwensis* and *T. papuae*, reptiles were successfully infected with an inoculum of 3000 larvae per animal (Pozio et al. 2004). This dose might have been greater than inocula used for catfish and tigerfish in this study although information on the weight of animals was not given. Previous studies in other poikilothermic hosts, experimentally infected with different *Trichinella* taxa, suggest that temperature plays an integral role in larval survival and/or development in the host (Asatrian, Movsessian & Gevorkian 2000; Cristeau & Perian 1999; Pozio et al. 2004). In this study, water temperatures in ponds where catfish were kept were lower than those reported by Tomašovičová (1981) and Moretti et al. (1997).

Temperatures recorded during tigerfish trials in this study were much higher than those reported in the studies conducted by Tomašovičová (1981) and Moretti et al. (1997) and compared favourably with the lower end of temperatures reported by Pozio and La Rosa (2005). Water temperature in the river was significantly higher compared to the experimental ponds for T_1_ (*p* = 0.002), T_2_G_1_ (*p* = 0.0002), T_2_G_2_ (*p* = 0.002), T_3_G (*p* = 0.04) and T_3_G_3_ (*p* < 0.0001), but not so for T_3_G_1_ (*p* = 0.07). This precludes any definitive conclusion concerning the influence of temperature on *T. zimbabwensis* larval development and survival in fish.

Previous studies also suggested several host-related factors for the failure of *T. zimbabwensis* to establish in fish and this includes: (1) lack of villi in the small intestine preventing larvae from anchoring to the intestinal wall and resist peristaltic movements, (2) the copious amounts of bile and mucous excreted in the intestine of fish, (3) low body temperature of the host, (4) quick passage of larvae through the intestine and (5) possible destruction of larvae by digestive processes (Moretti et al. 1997).

*Trichinella pseudospiralis* larvae have been reported to migrate without moulting from the gastrointestinal tract to the body cavity, organs and muscles of fish whilst *T. spiralis* larvae only migrated to the body cavity of fish (Tomašovičová 1981). *Trichinella britovi* larvae migrated without further development and were isolated from the gastrointestinal tract and body cavity of carp (*Cyprinus carpio*) and from the gastrointestinal tract of catfish (*Ictalurus melas*) (Moretti et al. 1997). In this study, no positive results could be obtained from the fish during the early stages after infection (day 1, 2 and 7 p.i.) in catfish or day 7 p.i. in tigerfish.

From an epidemiological perspective, the negative results from this study in catfish rule out the potential of this fish species as a host for *T. zimbabwensis*. Positive results from tigerfish, although interesting, are inconclusive on whether tigerfish could act as reservoir hosts for *T. zimbabwensis*.

## Conclusion

Results from this study suggest that *T. zimbabwensis* is unable to develop and establish in the African sharp tooth catfish, however, results from tigerfish suggest that some individual fish could, under very specific circumstances, maintain *T. zimbabwensis* larvae. It is not known whether the parasite can fully develop and reproduce in this host.

The paucity of information concerning the minimum infective dosage required to establish *T. zimbabwensis* infection, the potential influence of host (Kapel 1995; La Grange et al. 2013; Reina, Munoz-Ojeda & Serrano 1996; Soule et al. 1989) and parasite characteristics (Hurnikova et al. 2004; Kapel, Webster & Gamble 2005) and the potential influence of temperature on larval development and survival in fish precludes a general hypothesis that fish do not play any role in the epidemiology of *T. zimbabwensis*. Future research efforts should focus on additional tropical fish that cohabit with other known hosts of *T. zimbabwensis* in the GKNP to elucidate the role of any specific fish species in the epidemiology of *T. zimbabwensis*.

## References

[CIT0001] Asatrian A., Movsessian S. & Gevorkian A., 2000, ‘Experimental infection of some reptiles with *T. spiralis* and *T. pseudospiralis*’, Abstract book, The Tenth International conference on Trichinellosis, Fontaineblue, 20–24 August, p. 154.

[CIT0002] Bruton M.N., 1979, ‘The food and feeding behaviour of *Clariasgariepinus* (Pisces: Clariidae) in Lake Sibaya, South Africa, with emphasis on its role as a predator of cichlids’, *Transactions of the Zoological Society of London* 35(1), 47–114. 10.1111/j.1096-3642.1979.tb00057.x

[CIT0003] Cristeau G. & Perian A., 1999, ‘Experimental infestation with Trichinella in vipers’, Revista Romana de Parsitologia 9, 45.

[CIT0004] Dupouy-Camet J., 2000, ‘Trichinellosis: A worldwide zoonosis’, *Veterinary Parasitology* 93(3–4), 191–200. 10.1016/S0304-4017(00)00341-1 11099837

[CIT0005] Gardner B.R., 2017, *Anaesthesia of fresh water teleost fish for the private practitioner*, Copyright Reserved, South African Veterinary Association, 6 pages, viewed 17 February 2018, from http://www.cpdsolutions.co.za/Publications/org_publications_public.php?o=51.

[CIT0006] Gottstein B., Pozio E. & Nöckler K., 2009, ‘Epidemiology, diagnosis, treatment, and control of trichinellosis’, *Clinical Microbiology Review* 22(1), 127–145. 10.1128/CMR.00026-08 PMC2620635 19136437

[CIT0007] Hurníková Z., Dubinsky S., Mukaratirwa S., Foggin C.M. & Kapel C.M.O., 2004, ‘Infectivity and temperature tolerance on non-encapsulating *Trichinella zimbabwensis* in experimentally infected Red foxes (*Vulpes vulpes*)’, *Helminthologia* 41(4), 189–192.

[CIT0008] Justine J., Briand M.J. & Bray R.A., 2012, ‘A quick and simple method, usable in the field, for collecting parasites in suitable condition for both morphological and molecular studies’, *Parasitology Research* 111, 341–351. 10.1007/s00436-012-2845-6 22327319

[CIT0009] Kapel C., 1995, ‘*Trichinella* infections in Arctic foxes from Greenland: Studies and reflections on predilection sites of muscle larvae’, *Journal of Helminthology* 69(4), 325–330. 10.1017/S0022149X00014905 8583127

[CIT0010] Kapel C., Webster P. & Gamble H., 2005, ‘Muscle distribution of sylvatic and domestic *Trichinella* larvae in production animals and wildlife’, *Veterinary Parasitology* 132(1–2), 101–105. 10.1016/j.vetpar.2005.05.036 15979801

[CIT0011] La Grange L.J. & Mukaratirwa S., 2020, ‘Epidemiology and hypothetical transmission cycles of *Trichinella* infections in the Greater Kruger National Park of South Africa: An example of host-parasite interactions in an environment with minimal human interference’, *Parasite* 27(13), 1–12. 10.1051/parasite/2020010 32163031 PMC7067144

[CIT0012] La Grange L.J., Govender D. & Mukaratirwa S., 2013, ‘The occurrence of *Trichinella zimbabwensis* in naturally infected wild crocodiles (*Crocodylusniloticus*) from the Kruger National Park, South Africa’, *Journal of Helminthology* 87(1), 91–96. 10.1017/S0022149X12000089 22335961

[CIT0013] Moretti A., PiergiliFioretti D., Pasquali P., Mechelli L., Rossodivita M.E. & Polidori G.A., 1997, ‘Experimental infection of fish with Trichinella britovi: Biological evaluations’, in Ortega-Pierres G., Gamble H.R., Van Knapen F. & Wakelin D. (eds.), *Trichinellosis. Centro de Investigacion y Estudios Avanzados del Instituto Politecnico Nacional*, pp. 135–142, Mexico City.

[CIT0014] Mukaratirwa S., La Grange L.J., Malatji M.P., Reininghaus B. & Lamb J., 2019, ‘Prevalence and molecular identification of *Trichinella* species isolated from wildlife originating from Limpopo and Mpumalanga provinces of South Africa’, *Journal of Helminthology* 93(1), 50–56. 10.1017/S0022149X17001079 29168444

[CIT0015] Nöckler K. & Kapel C.M.O., 2007, ‘Detection and surveillance for *Trichinella*: Meat inspection hygiene, and legislation’, in Dupouy-Camet J. & Murrell K.D. (eds.), *FAO/WHO/OIE guidelines for the surveillance, management, prevention and control of trichinellosis*, pp. 69–97, World Organisation for Animal Health Press, Paris.

[CIT0016] Pienaar U. de V., 1968, *The freshwater fishes of the Kruger National Park*, pp. 1–20, National Parks Board, Pretoria.

[CIT0017] Pozio E. & La Rosa G., 2005, ‘Evaluation of the infectivity of *Trichinella papuae* and *Trichinella zimbabwensis* for equatorial freshwater fishes’, *Veterinary Parasitology* 132(1–2), 113–114. 10.1016/j.vetpar.2005.05.038 15982818

[CIT0018] Pozio E., Hoberg E., La Rosa G. & Zarlenga D.S., 2009, ‘Molecular taxonomy, phylogeny and biogeography of nematodes belonging to the *Trichinella* genus’, *Infection, Genetics and Evolution* 9(4), 606–616. 10.1016/j.meegid.2009.03.003 19460327

[CIT0019] Pozio E., Marucci G., Casulli A., Sacchi L., Mukaratirwa S., Foggin C.M. et al., 2004, ‘*Trichinella papuae* and *Trichinella zimbabwensis* induce infection in experimentally infected varans, caimans, pythons and turtles’, *Parasitology* 128(3), 333–342. 10.1017/S0031182003004542 15074882

[CIT0020] Reina D., Munoz-Ojeda M. & Serrano F., 1996, ‘Experimental trichinellosis in goats’, *Veterinary Parasitology* 62(1–2), 125–132. 10.1016/0304-4017(95)00835-7 8638385

[CIT0021] Roux F., Steyn G., Hay C. & Wagenaar I., 2018, ‘Movement patterns and home range size of tiger fish (*Hydrocynusvittatus*) in the Incomati River system, South Africa’, *Koedoe* 60(1), 1–13. 10.4102/koedoe.v60i1.1397

[CIT0022] Skelton P., 2001, *A complete guide to the freshwater fishes of Southern Africa*, 2nd edn., Struik Publishers, Cape Town.

[CIT0023] Soule C., Dupoy-Camet J., Georges P., Ancelle T., Gillet J.P., Vaissaire J. et al., 1989, ‘Experimental trichinellosis in horses: Biological and parasitological evaluation’, *Veterinary Parasitology* 31(1), 19–36. 10.1016/0304-4017(89)90005-8 2658299

[CIT0024] Tomašovičová O., 1981, ‘The role of fish in transfer and maintenance of Trichinellae under natural conditions’, *Biologia* 36(2), 115–125.

